# Preclinical *in vitro* evaluation of implantable materials: conventional approaches, new models and future directions

**DOI:** 10.3389/fbioe.2023.1193204

**Published:** 2023-07-27

**Authors:** Emilie Frisch, Lisa Clavier, Abdessamad Belhamdi, Nihal Engin Vrana, Philippe Lavalle, Benoît Frisch, Béatrice Heurtault, Varvara Gribova

**Affiliations:** ^1^ Université de Strasbourg, CNRS UMR 7199, 3Bio Team, Laboratoire de Conception et Application de Molécules Bioactives, Faculté de Pharmacie, Strasbourg, France; ^2^ Institut National de la Santé et de la Recherche Médicale, Inserm UMR_S 1121 Biomaterials and Bioengineering, Centre de Recherche en Biomédecine de Strasbourg, Strasbourg, France; ^3^ Université de Strasbourg, Faculté de Chirurgie Dentaire, Strasbourg, France; ^4^ SPARTHA Medical, Strasbourg, France

**Keywords:** biomaterials, biocompatibility, implants, 3D models, organoids

## Abstract

Nowadays, implants and prostheses are widely used to repair damaged tissues or to treat different diseases, but their use is associated with the risk of infection, inflammation and finally rejection. To address these issues, new antimicrobial and anti-inflammatory materials are being developed. Aforementioned materials require their thorough preclinical testing before clinical applications can be envisaged. Although many researchers are currently working on new *in vitro* tissues for drug screening and tissue replacement, *in vitro* models for evaluation of new biomaterials are just emerging and are extremely rare. In this context, there is an increased need for advanced *in vitro* models, which would best recapitulate the *in vivo* environment, limiting animal experimentation and adapted to the multitude of these materials. Here, we overview currently available preclinical methods and models for biological *in vitro* evaluation of new biomaterials. We describe several biological tests used in biocompatibility assessment, which is a primordial step in new material’s development, and discuss existing challenges in this field. In the second part, the emphasis is made on the development of new 3D models and approaches for preclinical evaluation of biomaterials. The third part focuses on the main parameters to consider to achieve the optimal conditions for evaluating biocompatibility; we also overview differences in regulations across different geographical regions and regulatory systems. Finally, we discuss future directions for the development of innovative biomaterial-related assays: *in silico* models, dynamic testing models, complex multicellular and multiple organ systems, as well as patient-specific personalized testing approaches.

## 1 Introduction

The number of implants and prostheses that are used to treat various diseases increases every year. However, implantation of biomedical devices is often followed by immune response to the implant, as well as by bacterial and fungal infections (Morais et al., 2010; VanEpps and Younger, 2016). Among healthcare-associated infections, about a half can be attributed to medical devices (Darouiche, 2004). Mortality due to these infections depends on the device type and can range from very low (<5%) for dental implants to impressive 25% for heart valves (Darouiche, 2001). Inflammation, which often occurs as a response to implantation, is another serious issue which can induce implant’s degradation and dissolution of surrounding tissue, leading to implant’s failure (Morais et al., 2010).

Although some of the infections can be successfully treated with antibiotics, bacterial resistance is another growing problem worldwide, which has been declared by World Health Organization as one of 10 biggest threats to global health (Laxminarayan et al., 2013). For the patients, it leads to longer duration of hospitalization, increased costs of treatment and higher morbidity. In this context, new implant materials and coatings that can decrease medical device-associated complications are urgently needed (Santos et al., 2016; Lebaudy et al., 2020).

However, bringing innovative materials to the market and making them available to the patients requires their thorough testing, which consists in preclinical and clinical studies. The approaches for preclinical testing will depend on the class of a medical device, but also on its intended use. For example, implantable materials’ evaluation will usually include *in vitro* classic monolayer cell culture (for instance for biocompatibility evaluation according to ISO-10993) and *in vivo* animal tests. Although well established, these two approaches have numerous drawbacks. On the one hand, classic monolayer cell culture does not reproduce physiological environment, which for the most tissues is 3D with spatially distributed biophysical, biochemical and mechanical cues. On the other hand, animal tests are extremely expensive, long and have significant ethical issues. In addition, animal physiology is, in most cases, significantly different from the human one, rendering the results not fully representative of the clinical conditions.

New 3D models, such as organoids or scaffold-based engineered tissues, are currently being developed for various applications, e.g., drug testing (Langhans, 2018; Jensen and Teng, 2020; Liu et al., 2021). Thus, patient-derived tumor organoids can allow better selection of the anticancer therapy (Calandrini et al., 2021), and 3D-printed cell-laden hydrogels were developed for chemotherapeutic drug screening (Gebeyehu et al., 2021). Other tissues of interest include β-cell spheroids for diabetes drug screening (Jesal et al., 2016), neuromuscular models (Osaki et al., 2020), or, recently, 3D culture models to study SARS-CoV-2 infectivity (de Melo et al., 2021). It is acknowledged that 3D environment is different from 2D one, as 3D models are required, for instance, to better mimic *in vivo* response to a treatment.

Despite this active development of 3D systems in drug delivery and in pathologies modelling, 3D tissue models are almost absent and only start to emerge in the field of biomaterials testing (Ren et al., 2019; Barker et al., 2020). This is very unfortunate, because many biomaterials are intended for use in a 3D environment, and 2D models cannot recapitulate the complexity of such environment.

Moreover, for functionality testing (e.g., antibacterial, anti-inflammatory), even more complex co-culture models are required that do not yet exist in biomaterials field. Only few 3D infection models have been developed. For instance, gastric organoids were used to study *Salmonella* and *Helicobacter pylori* infection, and human intestine-on-a-chip model was developed to investigate the effect of pathogenic bacteria (Shi et al., 2019). Inflammatory 3D models are also starting to emerge. Recently, biomimetic 3D models for investigating the role of monocytes and macrophages in atherosclerosis were reported (Garcia-Sabaté et al., 2020).

**FIGURE 1:**
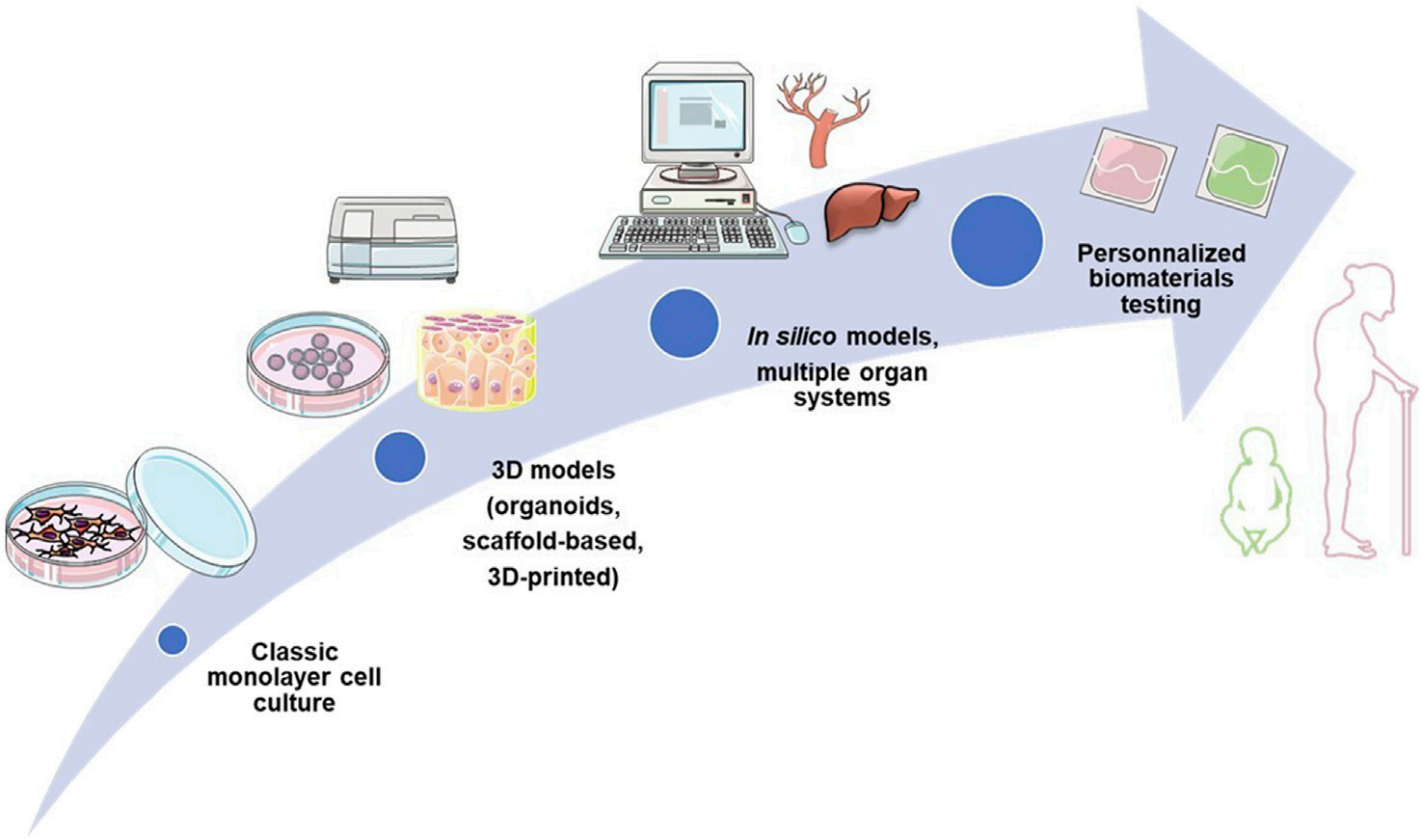
Evolution of biocompatibility testing: from classic monolayer culture to personalized testing approaches.

Here, we overview currently available preclinical methods and models for *in vitro* evaluation of new biomaterials. We firstly introduce conventional biocompatibility assessment approaches, which is a primordial step in new material’s development. The list of the tests is not exhaustive and is used to illustrate what currently used tests look like. For instance, we did not include biomechanical testing, as we focus on biological testing that implantable materials undergo. In the second part, the emphasis is made on the development of new 3D models and approaches for preclinical evaluation of biomaterials. The third part allows to highlight the main parameters, related or not to the sample, which will have to be taken into account for an optimal evaluation of the biocompatibility. We also rapidly overview differences in regulations across different geographical regions and regulatory systems (particularly USA vs. European). Finally, future directions for development of innovative biomaterial-related assays are described, such as *in silico* models, dynamic testing models, complex multicellular and multiple organ systems, and patient-specific personalized testing approaches (Figure 1).

## 2 Conventional approaches for preclinical testing of implantable materials

### 2.1 Biocompatibility testing

The notion of biocompatibility is a key issue. We are not unmindful of the scandal caused by Poly Implant Prosthesis breast implants at the end of the 2000 s. This accident involved breast implants made of a non-medical silicone whose poor quality considerably increased the risk of implant shell rupture. The danger was so high that the French medical regulatory authority removed these silicone implants from the market in 2010, leading other EU countries to follow suit. Although several studies have not revealed any particular toxicity of silicone, the causes of implant ruptures remain unknown. Bacterial infection or a strong immune response could be the cause of the contracture. While this scandal was not originally the result of a real biocompatibility problem but rather a deliberate attempt, it has become urgent to improve the evaluation of implants and biomaterials for medical and therapeutic applications to avoid similar accidents in the future (Bachour et al., 2018).

Biocompatibility describes the ability of a material to interact with live tissues without producing undesirable effects (cell death, hemolysis, inflammatory/foreign body response, mutations, etc.), Newly developed materials have to be thoroughly evaluated (Ghasemi-Mobarakeh et al., 2019) to guarantee their safety for the patients. To do so, ISO norms are used. The International Organization for Standardization (ISO) plays a crucial role in achieving global standardization by developing and publishing international norms. These norms cover a wide range of industries and sectors, from agriculture to healthcare, and more. By providing guidelines and requirements, ISO standards promote consistency and quality in products, services, and processes. This includes safety evaluation and assays, ensuring standardized approaches worldwide. Concerning the biomaterials intended for a medical purpose, one ISO standard known as ISO-10993 and entitled “Biological evaluation of medical devices” has been set up. This standard includes, for example, cytotoxicity evaluation (ISO 10993-5), or immunotoxicology testing of medical devices (ISO 10993-20). Other standards such as Organisation Européenne de Coopération Economique (OECD) guidelines have also been taken into consideration in biomedical devices evaluation, for instance OECD 471 for genotoxicity and mutagenicity testing.

#### 2.1.1 Cytotoxicity

Cytotoxicity is one of the most important criteria to assess during biocompatibility tests. Cytotoxicity testing is required for any new biomaterial and medical device development before animal experiments (Ghasemi-Mobarakeh et al., 2019). Main cytotoxicity testing methods are divided into 3 standardized categories following the state of the biomaterial: extract, direct contact and indirect contact tests (Sciences Assay Guidance Manual, 2004). Among them, numerous quantitative approaches can be selected to measure different criteria, including cell morphology, membrane integrity, adhesion, viability, proliferation, or cellular functions after contact with the material. The choice of the assay is very important, as well as the choice of the cellular model. Usually, well-known and common immortalized cell lines (HeLa or 3T3) are used. However, cellular model can also be selected according to the future medical applications of the biomaterials. For example, investigation on osteoblast cells is essential when the biomaterials are destined to be used as dental or orthopedic implants.

Dye assays such as Trypan blue assay and Neutral red uptake-based assay are simple methods based on molecules that are excluded or, on the opposite, are internalized by viable cells. The second subcategory is based on the biochemical measurement of the metabolic activity of living cells. Formazan-based methods like MTT [3-(4,5-dimethylthiazol-2-yl)-2,5-diphenyltetrazolium bromide] test and its derivatives, or lactate-dehydrogenase (LDH) release assay are commonly used in laboratories. Close to these techniques, fluorimetric assays like Alamar Blue™ and luminometric ATP-assays are also simple and trustable tests. Radioactivity-based assays such as (^3^H)-thymidine assay performed to measure cell proliferation are gradually abandoned to reduce radioisotopes utilization.

As previously described, several tests are available to assess cytotoxicity. Each assay tests a different aspect of the cell biology (metabolism, membrane permeability, etc.), and offers its own advantages and disadvantages summarized in Table 1.

**TABLE 1:** Summary of different benefits and drawbacks of main cytotoxicity assays.

ISO	Assays	Studied biological parameter	Benefits	Disadvantages
ISO 10993-5	Dye assays (Trypan Blue assays)	Membrane integrity and cell permeability	Cheap, rapid, simple	Counting errors, only dead cells are stained, small number of samples
	Metabolic-based assays (MTT or LDH release assays)	Activity of mitochondrial enzymes (succinate dehydrogenase)	Easy, safe, high reproducibility	Cell and activation state-dependent, overestimation of viability
	Fluorimetric assays (Alamar Blue)	Activity of mitochondrial enzymes (diaphorases)	More sensitive than MTT assays, relatively cheap, use of other technics at the same time	Fluorescence interference
	Luminometric ATP assays (CellTiter Glo^®^)	ATP synthesis ability	Really sensitive, quick, easy, less biological background	Limitation of the reproducibility
	Radioactivity-based assays [(^3^H)-thymidine or (^51^Cr) assay]	Cell proliferation	Sensitive, large number of samples	Radioactive and manual workload

Another criterion that can be checked when investigating cytotoxicity is cell death, through apoptosis or necrosis studies, for instance. Distinction between the type of death is important. In contrast to apoptosis, which is a so-called “clean death”, necrosis is often accompanied by an underlying inflammation related to cell lysis. This inflammation can lead, in case of biomaterials, to their destruction and to significant tissue damage. Thus, skin necrosis is a complication that often occurs after mastectomy, making breast reconstruction difficult. In a majority of cases, this necrosis ends in the removal of the implant. Better characterization and standardization of tests are therefore needed (Sue et al., 2018). Besides the assays already discussed above, apoptosis-specific methods have also been developed. For example, staining of apoptotic phosphatidylserine by Annexin V bound to a fluorochrome can be detected by confocal microscopy or by flow cytometry. Other techniques include notably deoxynucleotidyl transferase (Tdt)-mediated dUTP nick-end labelling (TUNEL) assay, a marker of apoptotic DNA fragmentation (Martínez et al., 2021) (Table 2).

**TABLE 2:** Summary of the main assays needed to assess the key biocompatibility parameters (cytotoxicity, hemocompatibility, inflammation, and genotoxicity).

General biological response	ISO or OECD standards	Biological subcategory response	Assays	References
Cytotoxicity	ISO 10993-5	Viability	Dye assays (trypan blue or neutral red uptake-based assays)	Özlem Sultan et al. (2017), Liu X. et al. (2018), Bernard et al. (2018)
			Metabolic-based assays (MTT or lactate-dehydrogenase release assays)	
			Fluorimetric assays (Alamar Blue)	
			Luminometric ATP assays (Celltiter Glo®)	
			Radioactivity-based assays ((3H)-thymidine assay)	
		Cell death	Apoptosis-detection assays (Annexin-V staining or TUNEL assay)	Sciences Assay Guidance Manual (2004), Martínez et al. (2021)
Hemocompatibility	ISO 10993-4	Thrombogenicity	ELISA	Bernard et al. (2018)
		Coagulation	Microscopy	
		Platelets	Flow cytometry	
			Prothrombin time assay or partial thromboplastin time assay	Ng (2009)
		Leukocytes	Blood cell counters	Nalezinková (2020)
		Hematology	Hemoglobinometer	Ranganathan and Gunasekaran (2006), Nalezinková (2020)
			Spectrophometry assays (Cyanmethemoglobin method)	
Inflammation and Foreign Body Reaction for implantable biomaterials	ISO 10993-6 ISO 10993-20	Blood plasma proteins adsorption	ELISA	Anderson et al. (2008), Bernard et al. (2018), Lock et al. (2019)
		Recruitment, activation and fusion of macrophages cells	qPCR	
			Immunohistochemistry	
			Microscopy	
			Flow cytometry	
			Phagocytosis assay (Cytoselect^TM^ 96-well phagocytosis assay)	Smith et al. (2010)
			Chemiluminescence assay (NO derivatives measurment)	Di Fenza et al. (2022)
		Fibrotic tissue formation	Fibrosis assay	Basu et al. (2011)
Genotoxicity	ISO 10993-3 OECD 471	Gene mutational assays	Ames test	Maron and Ames (1983), Gupta (2016)
			Mouse lymphoma gene mutation assay	
		Chromosomal aberration assays	*In vitro* micronucleus assay	Cervena et al. (2021)
		DNA damage-based assays	Single cell gel electrophoresis assay	Azqueta and Dusinska (2015)

#### 2.1.2 Hemocompatibility

Hemocompatibility is another important property required for any biomaterial’s marketing authorization (Table 2). This property mostly concerns thrombosis risk and coagulation problems. Three additional criteria including platelets, hematology and leukocytes activation are also added, bringing the number of categories needed to assess to five (Nalezinková, 2020). In order to meet all the requirements and due to the complexity of blood composition (erythrocytes, leukocytes, platelets and complement proteins), multiple tests must be performed. Together with a careful choice of the assay based on the type of blood/biomaterial contact (direct or indirect contact), the choice of the blood model and of the incubation method must also be taken into consideration (Weber et al., 2018). This is supported by a recent study performed by Block et al., which reveals that storage of the fresh blood had a significant influence on blood responses and therefore on hemocompatibility testing (Blok et al., 2016).

General tests, such as Enzyme-Linked-Immunosorbent-Assay (ELISA) or flow cytometry, are available for almost all of the five aforementioned hemocompatibility criteria. In addition, some assays are more specific and can be used in complement. For instance, thrombosis risk is particularly investigated when biomaterials are intended for cardiovascular applications. If we take the example of cardiac stents, those of the first generation had a particularly high associated risk of thrombosis. Nowadays, researchers are working on polymer-based coatings for better biocompatibility and better thrombosis risk prevention (Rudolph et al., 2015). While it is easier to test thrombogenicity using an *in vivo* model, some *in vitro* assays exist. They mostly include microscopy techniques, and more precisely Scanning Electron Microscopy (SEM) to check platelets adhesion, aggregation and morphological modifications (Bernard et al., 2018).

Microscopy is also a key method for coagulation evaluation. However, some tests such as prothrombin time assay or the partial thromboplastin time assay can also be used to measure the extrinsic and the intrinsic coagulation pathways, respectively (Ng, 2009).

To complete the evaluation of the main categories required in the ISO standards, platelets, leukocytes and hematology evaluation can be done with conventional blood cell counters or with more specific platelet function analyzers (PFA-100) able to monitor platelets aggregation and to measure the closure time (Nalezinková, 2020).

Depending on classification and official standards, hemolysis has not always been a mandatory sub-criterion essential for biomaterials evaluation. However, since 2017, the International Organization for Standardization has strongly reinforced the assessment of hemolysis. Before this date, hemolysis tests for biomaterials were not required, but only widely encouraged (ISO 10993‐4). To evaluate hemolysis, total hemoglobin concentration or plasmatic hemoglobin concentration in blood can be measured using hemoglobinometer or specific spectrophotometry tests, such as the cyanmethemoglobin method (Ranganathan and Gunasekaran, 2006).

#### 2.1.3 Inflammation

Inflammation is a defense response of the body to biological, physical or chemical damage. In response to implantable materials, inflammatory reaction called foreign body reaction (FBR) usually occurs (Table 2). Although this is a normal reaction of the body to a foreign material, a chronic response can happen, resulting, in the worst case, in implant failure (Saleh and Bryant, 2017).

**FIGURE 2:**
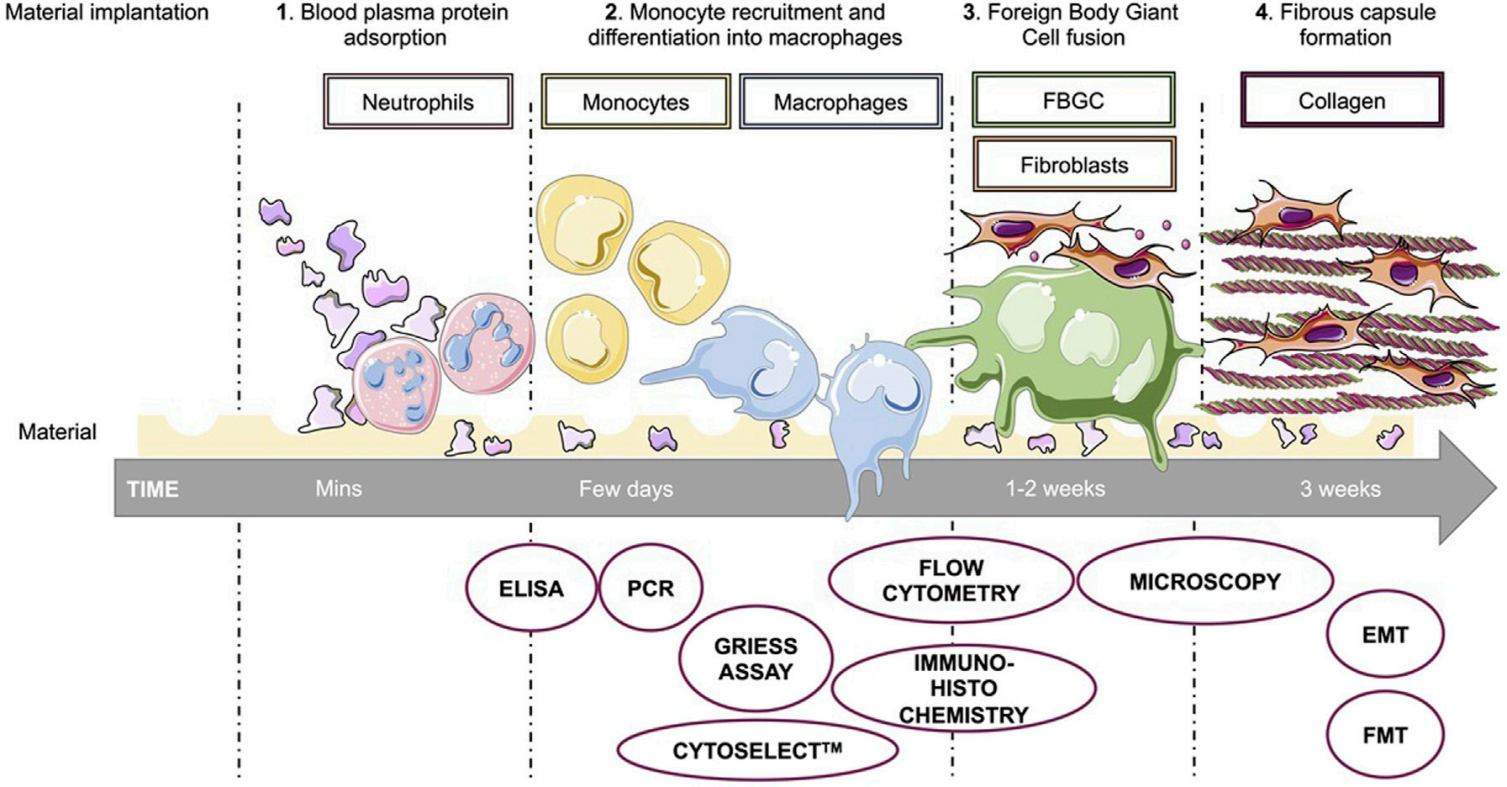
Foreign body reaction to biomaterial implantation and respective evaluation. Quickly after the implantation of the biomaterial, blood plasma proteins are adsorbed on the surface of the material. Their presence on the implant allows recruitment of leukocytes, including neutrophils and followed by monocytes. Differentiation of monocytes into macrophages and then their fusion into foreign body giant cells (FBGC) finally lead to recruitment of fibroblasts and to formation of a fibrous capsule around the implant, leading to chronic inflammation. Each of the foreign body reaction steps can be assessed through more or less specific assays. Enzyme-linked immunosorbent assay (ELISA), polymerase chain reaction (PCR), microscopy or flow cytometry are common methods to study biocompatibility. In addition to these, there are more specific tests such as Griess assay for nitric oxide secretion, Cytoselect™ 96-well phagocytosis assay to study the activation of macrophages, or Epithelial to Mesenchymal Transition (EMT) and Fibroblast to Myofibroblast Transition (FMT) to study the fibrotic tissue formation.

FBR is characterized by 4 different steps, starting with blood plasma protein adsorption on the materials surface. Enzyme-linked immunosorbent assay (ELISA) is one of the simplest tests used to quantify specific proteins and especially the amount of albumin, fibrinogen or complement cascade C3a and C5 proteins. This last point can also be achieved through screening assays, such as hemolytic complement activity assay (Costabile, 2010). This phase is followed by monocyte recruitment and differentiation into macrophages, and then by their fusion into giant cells. Fibroblasts are usually the last cells to be recruited, and contribute to the creation of a persistent fibrous capsule around the material during the final step. This encapsulation isolates the implant from the rest of the body and leads to the establishment of chronic basal inflammation (Anderson et al., 2008; Lebaudy et al., 2020) The key steps of the FBR are developed in Figure 2.

FBR is poorly studied *in vitro* because of the number of factors involved and the difficulties to elaborate a complex inflammatory model. The official standards do not recommend specific tests dedicated to the *in vitro* study of FBR, as its evaluation is mainly carried out on animal models. Unfortunately, reproducibility of such experiments is unsatisfactory and questions the relevance of animal models to study human pathologies and reactions (Wang et al., 2017). However, as monocytes and macrophages are the main cell types implicated in FBR, it is easier to just focus on them. To this end, primary cell culture or cell lines as the murine RAW 264.7 macrophages or the human THP-1 monocytes are generally used (Bernard et al., 2018).

Assessment of the recruitment and activation of monocytes and macrophages around the implant is usually done through the *in vitro* study of inflammatory markers expressed by the cells. For this purpose, common laboratory methods are generally implemented. They may include flow cytometry, ELISA, or polymerase chain reaction (PCR) to combine evaluation of proteins and gene expression (Lock et al., 2019). Other more qualitative techniques have also been adopted, such as protein microarray or confocal microscopy (Lock et al., 2019). Walschus et al. have used, for their part, an immunohistochemistry approach to identify macrophages and antigen-presenting cells in the proximity of a titanium-based implant (Walschus et al., 2011).

During the acute phase of the FBR, activated macrophages acquire and develop new mechanisms of action, such as production of reactive oxygen species (ROS), nitric oxide (NO), release of inflammatory cytokines or phagocytic activity. Recent methods to study phagocytosis are based on immunoglobulin G opsonised sheep red blood cells like the CytoselectTM 96-well phagocytosis assay. Chemical Griess assay or chemiluminescence measurement are usual approaches used to detect the presence of NO derivatives (nitrite ion NO2-) and ROS, respectively (Lock et al., 2019).

The chronic phase of FBR begins with fibrosis of the peripheral tissue around the implant. This stage is characterized by fusion of macrophages into polynucleated foreign body giant cells (FBGC) and recruitment of fibroblasts. Flow cytometry, immunohistochemistry and confocal microscopy are a good way to assess macrophages fusion into giant cells or fibrotic tissue formation (Anderson et al., 2008). Basu et al. developed a method to test fibrosis using Epithelial to Mesenchymal Transition (EMT) or Fibroblast to Myofibroblast Transition (FMT) characterization (Basu et al., 2011). The main assays used to evaluate the FBR are shown in Figure 2.

#### 2.1.4 Genotoxicity

Genotoxicity is the ability of chemicals or materials to induce genetic damage, which can eventually cause cancer (Cvetković et al., 2018). If we consider the example of textured breast prostheses, it appears that they could be associated with a higher risk of anaplastic large cell lymphoma development. Although the pathogenesis of this kind of cancer is not well understood, some studies suggest that the implant-associated lymphoma is often related to JAK/STAT pathway activating mutations (Laurent et al., 2020). With this case in mind, genotoxicity seems to be an important feature to investigate in any biocompatibility assay before marketing authorization (Table 2).

Genotoxicity assays may be classified into 2 subcategories: gene mutational assays and chromosomal aberration assays (Cvetković et al., 2018). Ames test belongs to the first category and is used to study the biomaterials’ mutagenic ability to reverse metabolic mutations in *Salmonella typhimurium* bacterial strains (Maron and Ames, 1983). In a study by Kumari et al., the authors used this method to investigate the mutagenicity effect of plant extract-based materials for a new dental atraumatic restorative treatment (Kumari et al., 2019). A similar test could be done with eukaryotic cells using mouse lymphoma gene mutation assay, which consists in measuring the resistance of potentially mutant cells to a lethal drug (Gupta, 2016).

The second category of tests comprises chromosomal aberration assays and is mainly applied to detect structural and chromosomal number abnormalities induced by chemical agents and medical devices. As described in a recent study written by Cervena et al., *in vitro* micronucleus assay could be used to assess the genotoxic potential of some metallic nanomaterials through the evaluation of chromosomal separation during mitosis (Cervena et al., 2021).

Some genotoxicity tests are not required by ISO standards, but can be used in addition—for example, DNA damage-based assay known as single cell gel electrophoresis assay (Azqueta and Dusinska, 2015). In this case, alternative certified testing methods delivered by other international organizations has been used. OECD 471 guidelines are considered as references for genotoxicity evaluation.

## 3 Advanced approaches for evaluation of materials safety and functionality

### 3.1 Emergence of 3D models

**FIGURE 3:**
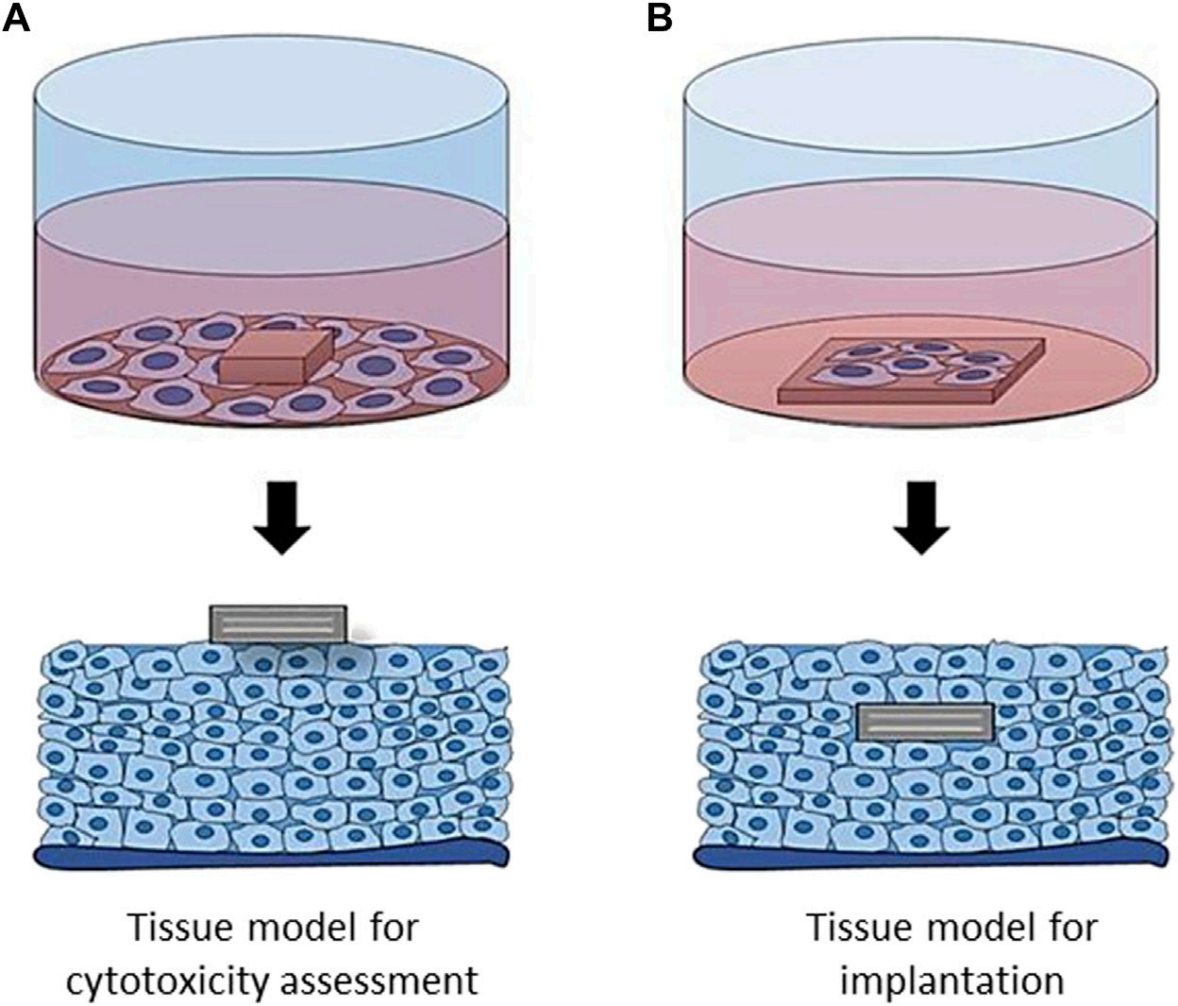
Evaluation of materials cytotoxicity **(A)** and cell adhesion **(B)** in 2D and 3D environment. In 2D, only one cell layer is in contact with the material, which may lead to an overestimation of its toxicity, as compared with 3D model. In a similar way, material interaction with the surrounding tissues is more complex in 3D, with the cells surrounding the implant from all the sides.

First cytotoxicity tests during biomaterials evaluation are usually conducted on cell monolayers, allowing a fast evaluation of material’s cytotoxicity. However, this model is far from physiological conditions: live tissues are three-dimensional, multi-layered and composed of several cell types. For this reason, even if a potential therapeutic molecule is found cytotoxic after a conventional 2D assay, it may appear less toxic in a 3D model, where the outer cell layer can protect inner layers from direct exposure (Figure 3A). In addition, different cell types have variable sensitivity towards the same molecules, so using only one cell type to assess material’s toxicity may not be optimal.

What about implantable materials? While interactions with the surrounding tissues are of the highest importance, their *in vitro* evaluation methods remain scarce. Here again, the *in vitro* environment is far from being physiological, since materials are not in relation with the whole cellular environment of a complex organism (Figure 3B).

As already discussed above, the development of more physiological tissue models became a priority. 3D models, such as organoids or scaffold-based engineered tissues, are already being developed for various applications, from drug testing (Jensen and Teng, 2020) to meat production (Dong-Hee et al., 2020). However, for new biomaterials testing, 3D tissue models are almost absent and only start to emerge.

### 3.2 Existing models

**FIGURE 4:**
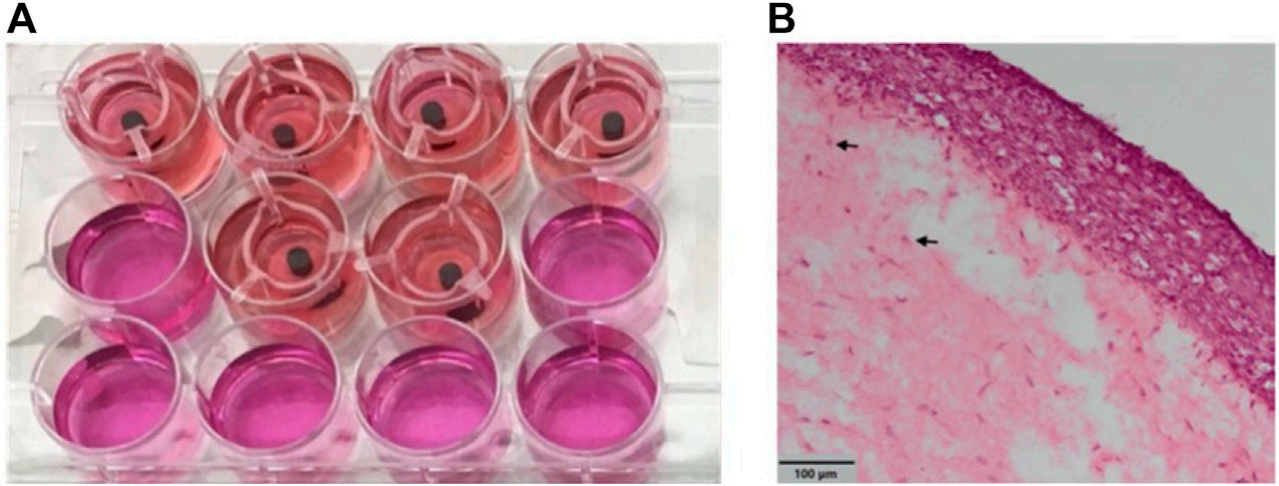
Engineered oral mucosal models with inserted implant materials **(A)** and histological views of oral mucosal models **(B)**. Adapted from (Barker et al., 2020). Copyright 2020, the Authors. Published by MDPI, Basel, Switzerland.

Due to the acknowledgment of their need by the scientific community, some 3D cellular models already exist for several years. In a pilot study by Barker et al., human oral mucosal model was used to study dental implant attachment to the engineered tissue (Barker et al., 2020). To do so, the researchers assembled human oral fibroblasts, OKF6/TERT-2 keratinocytes, and THP-1 monocytes into a 3D oral mucosal model inside tissue culture inserts. Different metal, ceramic, and polymer implant pieces were inserted into tissue-engineered oral mucosa following by a Ø 4 mm punch biopsy (Figure 4A). Implant and soft tissue attachment were then assessed using histology and scanning electron microscopy (Figure 4B). According to the authors, this model has a potential to be used for visualization and quantification of implant soft-tissue attachment.

Another example in the dental domain relates to periodontitis. Described as the inflammation and the destruction of the tooth-surrounding membrane and especially of the periodontal ligament, periodontitis requires specific cares. Regenerative solutions for periodontitis’ treatment are being developed, and require in particular new polymeric biomaterials that can serve as a matrix for tissue regeneration. In this context, Koch et al. have worked on the development of an injectable fibrillar biomaterial based on self-assembled peptides as a scaffold (Koch et al., 2020). To evaluate this new material, the authors designed a 3D *in vitro* periodontal model. The model is composed of two compartments: one compartment to simulate the periodontal ligament, which is composed of a hydrogel containing the ligament cells, and the other compartment based on human dentin which will receive the biomaterial. Thus, this model enabled the study of key parameters for evaluating tissue regeneration as ligament cells migration to the peptide matrix, viability of periodontal ligament fibroblasts and deposition capacity of ECM proteins. The authors are currently evaluating their peptide-based scaffold *in vivo*.

Thus, there are already some examples of preclinical evaluation of implantable biomaterials found in the literature, but they are not yet widespread. Other models, more particularly spheroids, organoids, have a potential to be used for biomaterials assessment, even if they haven’t been designed for this purpose. Their use as a model for the biomaterials’ evaluation will be developed further.

### 3.3 Promising models for the evaluation of biomaterials

Vascular medicine and cardiology are key fields for 3D *in vitro* models development and use. Kupfer et al. aimed to develop a complex 3D bioprinted heart model that could be used as an *in vitro* model or as a therapeutic option (Kupfer et al., 2020). In order to 3D print a heart, they first developed a bio-ink that promoted stem cells proliferation and differentiation into cardiomyocytes. This resulted in an organoid physiogically and electro-mechanically similar to the human heart, with two functional inlet and outlet chambers. As the authors point out, this technical advance could therefore serve as a testing bed for medical devices.

Bioprinting is also used to design 3D blood vessels. To improve cell survival during bioprinting, new processes for optimal material preparation need to be developed and optimized. Liu et al. have developed for instance the Multinozzle Multichannel Temperature Deposition System for tissue engineering and organ regeneration (Liu H. et al., 2018). Their innovative system allowed extrusion of different bioinks making the tubular structure representative of the blood vessel, but also incorporation of encapsulated biomolecules for cells protection.

To continue in the vascular field, Wimmer et al. have developed self-organized 3D human blood vessel organoids from human pluripotent stem cells (hPSC) (Wimmer et al., 2019). These blood vessel organoids have been used to study diabetic vasculopathy by exposing them to hyperglycaemia and inflammatory cytokines *in vitro.* Briefly, to generate these organoids, Wimmer et al. induced the differentiation of hPSC cells aggregates on a 3D collagen matrix, resulting in blood vessel networks of endothelial cells and mural cells in 11 days.

These 3D blood vessel models, although not intended for this purpose, could be used for the pre-clinical evaluation of new vascular biomaterials. The urgent need to prevent the coagulation cascade, as stent implantation required taking anticoagulant medication for life, has made essential the evaluation of new biomaterials intended for a cardiovascular use. In this context, vascular organoids could help to develop new implant coating to prevent coagulation, thus improving stent longevity and patients’ quality of life.

Bone organoids are another example as a preclinical model application. In a review concerning the progress and prospects of 3D bone models, Chen et al. mention the potential of bone organoids (Chen et al., 2022). According to them, it would be possible to test biomaterials for bone implants (ceramic, metal or polymeric) and thus limit the sacrifice of animals for *in vivo* evaluation by mimicking the best human internal environment. Several examples are mentioned, including the fabrication of a woven bone organoid (Akiva et al., 2021). This study reports the differentiation of human bone marrow stromal cells, seeded on silk fibrin scaffolds, into a functional self-organizing 3D co-culture of osteoblasts and osteocytes. By mechanically stimulating the cells, they produced an extracellular collagen matrix that could be mineralized under biological control.

3D skin models are also of great interest, as various skin diseases or burns can lead to significant skin loss. In addition, skin models are required to replace allergy testing on animals in cosmetics, which is a huge ethical issue. Hence, the need for 3D *in vitro* skin models is urgent and crucial.

**FIGURE 5:**
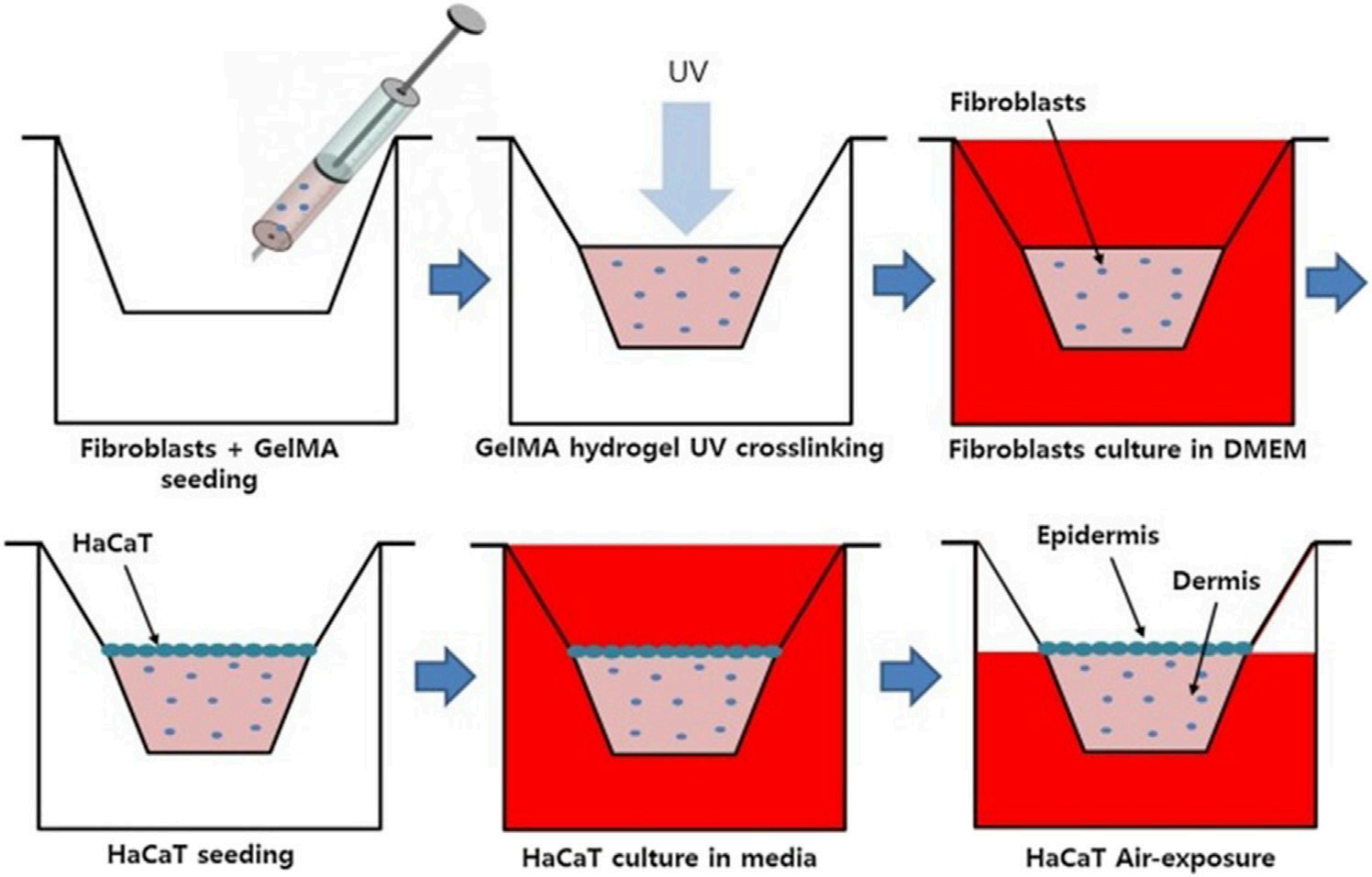
Schematics showing the experimental steps for construction of *in vitro* 3D skin model using gelatin methacrylate (GelMA) scaffold Adapted from (Kwak et al., 2018). Copyright 2018, Elsevier.

By now, few skin models are commercialized, even if their improvement remains fundamental. In most cases, these models consist of an artificial dermis composed of a biopolymer sponge seeded with fibroblasts to form a reconstituted, stratified and differentiated epidermis. The majority of the actual models use collagen as a matrix for cell development, as it is the most common protein found in the extracellular matrix. This is the case, for example, of the commercial skin model EpiDermFT™. However, collagen has a low mechanical resistance, so it is necessary to find alternatives. Kwak et al., in their study, sought to replace collagen with a gelatin cross-linked methacrylate hydrogel, which has better mechanical properties (Kwak et al., 2018). In addition, they developed a 3D skin model where fibroblasts were encapsulated in the gelatin-methacrylate gel and then cultured for a few days to build up the dermis. Keratinocytes (HaCat) were then seeded on the gel and cultured (Figure 5). The results of their study revealed that fibroblasts preferred a gelatin-methacrylate scaffold of a low polymer concentration which is less rigid and more porous, while keratinocytes favored a rigid and higher polymer concentrated gelatin-methacrylate matrix with a stronger mechanical resistance. The use of this hydrogel as a scaffold is therefore possible to build a 3D skin model, but only after an optimization of the microenvironment since the different cells in the skin seem to have different optimal environmental conditions.

Healthy physiologically 3D organ models are not sufficient to mimic actual biomaterials implantation. Indeed, in many cases, an inflammatory environment could be found around the medical device site implantation. Developing inflammatory 3D models is therefore legitimate to evaluate biomaterial’s ability to prevent deleterious body responses. Thus, Pupovac et al. published a review on immunocompetent skin models which more adequately mimic the skin (Pupovac et al., 2018). They described different methods for developing these models. They form complex multicellular models containing immune cells such as Langerhans cells, macrophages or T lymphocytes. Smith et al., for their part, successfully created a 3D skin model reproducing the inflammatory microenvironment of diabetic ulcers (Smith et al., 2021). These wounds are very difficult to treat so this new type of model could provide a better understanding of this disease and also serve as a testing platform for new drugs. Briefly, the model was created by co-culturing monocytes and fibroblasts from diabetic foot ulcer patients. Two monocyte incorporation techniques were tested to mimic the inflammatory microenvironment: the first one involved the incorporation of polarized pro-inflammatory M1 macrophages while the second required the incorporation of non-polarized monocytes from diabetic patients directly with the fibroblasts. The first results suggested that the differentiation of monocytes into macrophages was possible within the 3D skin model. Indeed, these macrophages had a cytokine secretory pattern similar to pro-inflammatory M1 macrophages phenotype found in diabetic foot ulcers.

To conclude, 3D models for biomaterials evaluation are extremely scarce, and we believe that it’s time to pay more attention to the development of advanced *in vitro* models for evaluation of new materials’ biocompatibility and functionality. However, whatever the models will be, we will be quickly confronted to many questions in relation with the sample itself such as its selection, methods of preparation and evaluation. Below, we discuss parameters to consider for medical device biocompatibility testing in an optimal way.

## 4 Optimization of medical device biocompatibility testing

**FIGURE 6:**
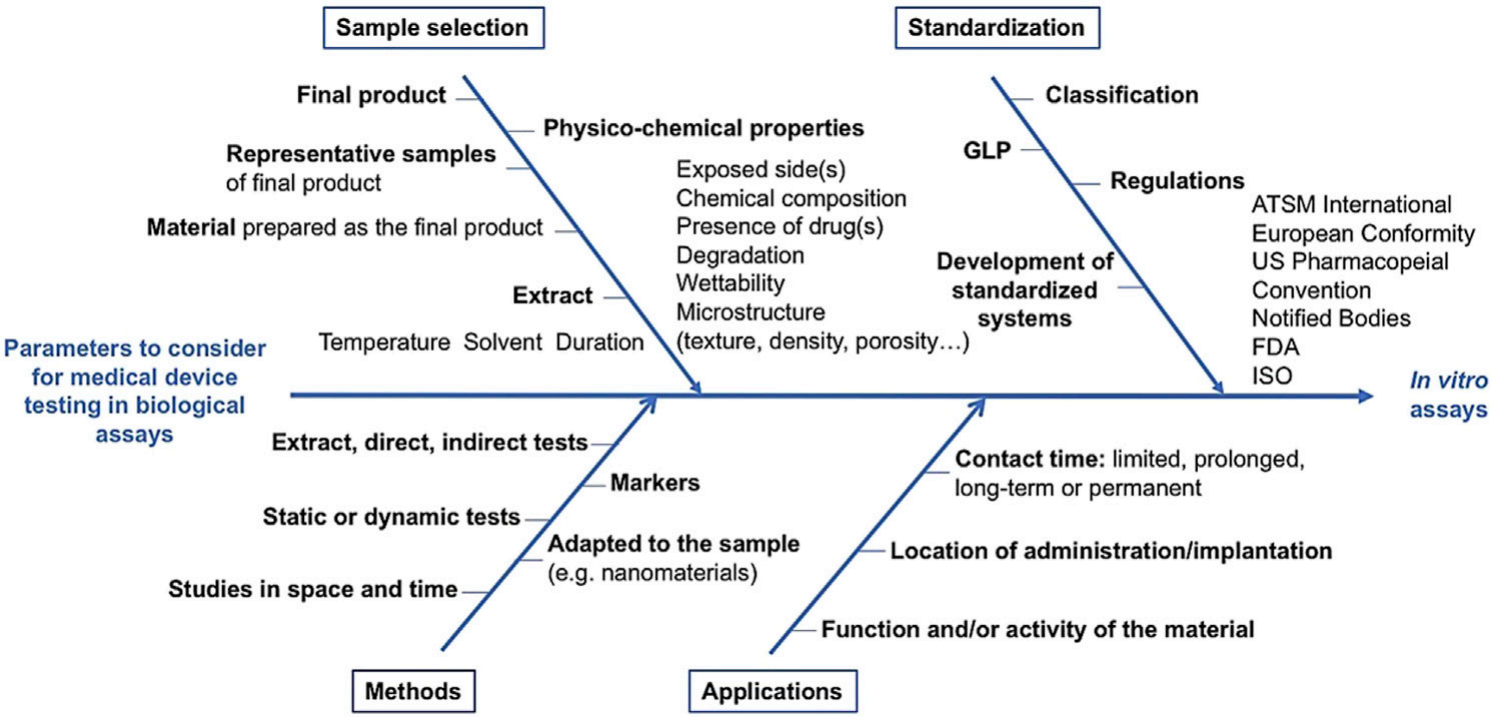
Parameters to consider for medical device testing biocompatibility. Main parameters have been classified into 4 categories related to the sample selection, testing methods, their applications and standardization.

The ISO 10993-12 norm specifies requirements and gives guidance on sample preparation procedures in biological test systems. However, multiple parameters able to influence final conclusion have to be considered for medical device testing in biological assays. Figure 6 distinguishes four non-exhaustive categories of parameters to take into account for biocompatibility studies (*i.e.*, sample selection, methods, applications and standardization), which are of prime importance and are discussed below.

### 4.1 Sample selection

Sample selection for biocompatibility studies is essential. ISO 10993-12 indicates that evaluation of biomaterial devices may not be limited to the final product, but that representative samples of the final product, material prepared as the final product, or extracts can also be used.

The final product corresponds to the complete device and is directly evaluated when it is technically possible. It has to be done after all steps of manufacturing, including, in particular, the steps of packaging and sterilization. However, even with the final and complete device, questions may arise, for example, in relation with the side exposed to the cells during the assay. Indeed, the controlled construction of bifacial biomaterials is a strategy to mimic anisotropic elements of the organism, such as skin layers or blood vessels (Im et al., 2018). The difference of properties as a function of the part of the sample can also be caused by the production process of biomaterials (Lucarelli et al., 2010). Those differences are not always fully studied and described before the evaluation of the sample. Whatever the reason, the method to evaluate the biocompatibility has to take into account potential differences between various faces in contact with biological matter (e.g., blood plasma, cells). When the device can’t be tested as a complete product, representative parts of the final device or materials prepared as the final product can be evaluated. Parts such as joints or coatings have to be proportionally represented. Finally, extracts from final devices (after packaging, sterilization …) can also be evaluated. Extraction conditions usually simulate physiological environment mimicking the clinical use (ISO 10993-18). In parallel, extraction in extreme conditions (increased temperature, organic solvent, long contact time) also brings information about the potential toxicity of substances which are likely to migrate from the device under degradation conditions. Polar, as well as non-polar, solvents can be used for extraction. In general, the fluid recovered is tested without buffering, dilution or filtration (Navarro et al., 2008). The extracts can also be characterized in terms of pH, osmolarity or ions content, in order to make possible relations between the results and the extract composition and properties (Costantino et al., 2020).

Chemical composition of the material, along with its structure, are also essential parameters to consider before the study, especially in case of new materials. Numerous physicochemical properties such as surface composition, surface degradation, hydrophilic-hydrophobic character, wettability, surface free energy, topography, stiffness and competitive protein binding have been demonstrated to be of key importance (Bernard et al., 2018; Jurak et al., 2021). For instance, surface characteristics are extremely important for thrombogenicity evaluation of medical devices in contact with the blood (Sarode and Roy, 2019). Therefore, there’s a need of morphological and topographical material characterization before evaluation (Liu C. et al., 2018). And in case of products containing active pharmaceutical ingredients, it is recommended that tests involving final products and drug-free products were performed.

Finally, degradation of the material can occur during its use, and the consequences must also be studied in biocompatibility assays. ISO 10993-13 to 15, specific to non-resorbable materials, describe identification and quantification of the degradation products of polymers, ceramics and alloys, respectively. Two kinds of degradation test methods are described corresponding to real-time testing and accelerated aging test. While the former provides sufficient information, the latter is not mandatory, depending on the expected duration of use of the medical device. The sample preparation has to be in accordance with ISO 10993-12. Since several decades, biodegradable/absorbable materials have been widely developed. These materials are particularly interesting for regenerative medicine, where they aim to trigger tissue regeneration, before they fully degrade inside the body. As a consequence, decomposition/degradation of the material, whether intended or not, may occur during use in human physiological environment, which imply some original conditions of biocompatibility evaluation, as degradation products may interact with the biological systems (Liu C. et al., 2018). In conclusion, numerous parameters have to be taken into account upon sample selection, to fully represent the complexity of the final device in all its states and conditions.

### 4.2 Choice of the methods for biocompatibility evaluation

Testing various samples under all possible conditions to assess biocompatibility can provide maximum insights on interactions of the material with biological system, for patient safety. However, it would be too expensive and time consuming, so only few samples are usually studied.

In terms of cytotoxicity evaluation, ISO 10993-5 leaves the choice between extract, direct or indirect contact tests. The parameters to consider orientating the choice are the nature of the sample, the potential site of use, and the nature of the use [ISO 10993-1 (Reeve and Baldrick, 2017)]. Furthermore, as materials can evolve, as well as interactions with the biological environment can trigger modifications (e.g., adsorption of various proteins), the samples need to be studied in space and time (Othman et al., 2018). *In vitro* static methods are mainly used, even if dynamic models are very interesting for specific materials such as mechanical circulatory support, extracorporeal membrane oxygenators, hemodialyzer, etc., A simple agitation of media over the surface of the material can be more relevant to *in vivo* conditions, however, true dynamic models are more appropriate for longer incubation periods.

Next, suitable tests and markers have to be chosen to measure the biological response. For instance, in thrombosis testing, 13 common markers are suggested by ISO 10993-4 (Sarode and Roy, 2019). The choice of the tests is also directly related to the sample. As example, evaluation of submicron or nanosize components is the subject of ISO 10993-22, including the evaluation of nanoobjects generated as products of degradation, of wear or of mechanical treatment processes from medical devices that are manufactured without using nanomaterials. Those materials have specific behavior, meaning that particular attention should be paid to nanomaterials. Indeed, their physico-chemical properties are different from those of bulk form and their interaction with the physiological environment is greatly increased due to i) increase of specific surface in contact with the cells and the physiological medium, ii) high diffusion around and inside the cells and iii) ability to directly interact with proteins. From a technological point of view, nanomaterials therefore imply to use specific characterization techniques compared to conventional ones. Furthermore, nanomaterials themselves should also not interfere with the chosen methods of evaluation. (Zor et al., 2019; Kyriakides et al., 2021).

### 4.3 Influence of the applications of the devices

To evaluate biocompatibility, the conditions of use of the device/biomaterials in the organism are paramount. Contact time can influence the risk for the patient, that’s why ISO 10993-1 define 3 categories. If the contact is less than or equal to 24 h, it is considered as limited contact. Greater than 24 h and up to 30 days corresponds to a prolonged contact. Finally, more than 30 days correspond to a long-term or permanent contact. Moreover, surface devices, external communicating devices and implant devices are distinguished. These different categories are taken into account to determine the type of tests to be carried out (FDA). As an example, for limited contact with a surface device, 3 biological endpoints are required for a mucosal membrane assessment (cytotoxicity, sensitization and irritation, or intracutaneous reactivity) compared to 7 for prolonged contact (the first three plus acute systemic toxicity, material-mediated pyrogenicity, subacute toxicity and implantation).

As an example, for bone graft substitute, Saos-2 human osteoblasts were cultured on discs composed of a mixture of hydroxyapatite and tricalcium phosphate. The authors analyzed cell cycle, integrity of the plasma membrane, cell viability, endothelial nitric oxide synthase expression, activity and mitochondrial membrane potential to define the biocompatibility (Alcaide et al., 2009). Giacomino et al. preferred using murine osteoblast precursor cell line (IDG-SW3) to study the biocompatibility of endodontic bioceramic sealers. In their work, viability was determined by luminescence assay based on ATP quantification (CellTiter-Glo^®^), but in addition to viability, osteoblastic differentiation and function were investigated (Giacomino et al., 2019). Comparison of these two works shows that the choice of the tests and the methods also depend on the site of application, as well as the function of the material. Safety and performance of a biomaterial or medical device are closely related, and function can also orientate the biocompatibility tests to be performed.

### 4.4 Regulation and standardization

In the dynamic landscape of regulatory affairs, it is crucial to stay well-informed about the latest standards and guidelines. This responsibility, known as regulatory watch or regulatory intelligence, is of utmost importance. However, it is also a challenging task due to the inconsistent and dissimilar evolution of regulations across different geographical regions and regulatory systems. These disparities can pose significant obstacles in the development of medical devices intended for use in diverse global markets and can significantly impact the progress of biocompatibility assessment studies (Reeve and Baldrick, 2017).

In the United States, healthcare products are regulated by the Food and Drug Administration (FDA). The FDA consists of various centers that oversee specific areas of regulation in accordance with the Federal Food Drug & Cosmetic Act (FD&C Act). Three major centers within the FDA are responsible for evaluating healthcare products. The Center for Devices and Radiological Health (CDRH) focuses primarily on regulating medical devices, including biomaterials, and assess their safety and effectiveness. The Center for Biologics Evaluation and Research (CBER) oversees the regulation of biological and related products including blood, vaccines, allergenics, tissues, and cellular and gene therapies. The Center for Drug Evaluation and Research (CDER) regulates over-the-counter and prescription drugs, including biological therapeutics and generic drugs. While CDRH is in most cases responsible of assessment of medical devices, CBER and CDER may participate in the evaluation of certain medical devices, such as those used in blood banks to produce biologics, which are regulated by CBER under the Medical Device Amendments of 1976 to the FD&C Act. Another instance is the evaluation of combination product (Drug-Device) where CDER and CBER may be assigned jurisdiction by the Office of Combination Products (OCP).

The FDA adopts a risk-based approach to evaluate medical devices, including biomaterials, considering the product’s nature and intended use (and sometimes indications for use). The levels of risk are categorized as low (or Class I), medium (or Class II) and high level (or class III). Based on their risk level and predicate status, medical devices have three major regulatory pathways to obtain approval: Premarket Approval (PMA), *De Novo*, and 510(k).

However, there are also special regulatory pathways intended to expedite medical device regulatory review. In December 2016, The US congress authorized the Food and Drug Administration (FDA) Breakthrough Devices Program (BDP) to replace the two previously existing special pathways: the Priority Review Program and the Expedited Access Pathway Program (EAP). This CDRH Innovation’s initiative aims to facilitate the development and availability of innovative devices for treating or diagnosing life-threatening or irreversibly debilitating human diseases or conditions. The program offers several benefits and features to eligible devices that meet specific criteria, including being breakthrough technologies, having no approved alternatives, offering significant advantages over existing options, and being in the best interest of patients.

The devices accepted into the BDP can utilize various features of the program These include Sprint discussions, which involve direct collaboration between device manufacturers and the FDA to promptly address any issues within an agreed-upon timeframe. The program also includes a Data Development Plan, which is a collaborative document between FDA and device sponsor outlining expectations for data collection during the premarket and post market phases to facilitate efficient FDA device review. Additionally, there is a Clinical Protocol Agreement, which is a written agreement between the FDA and the device sponsor that establishes clinical protocols before conducting studies. Also, Regular status updates in the form of scheduled discussions between the FDA and the device sponsor to provide general progress updates. Moreover, devices accepted into the BDP receive priority placement in the FDA’s review queue, expediting the review process through Priority Review (Johnston et al., 2020).

In Europe, the European Medical Device Regulation (EU MDR 2017/745) was enforced in May 2017 to regulate medical devices within the European Union (EU). Notified Bodies (NBs), which are private for-profit organizations, have the responsibility of evaluating medical devices in the EU. The NBs are designated by the European Union member states, based on relevant requirements, to carry out conformity assessment. The current list of Notified Bodies can be accessed through The NANDO website (New Approach Notified and Designated Organizations).

The primary criterion for approving a medical device in the EU, including biomaterials, is that its benefits outweigh its risks and that it performs as claimed. Once a medical device is approved, it is granted a CE mark (a French acronym for “European conformity”), indicating its compliance with EU relevant regulations. Only then can it be made available in the European market.

The evaluation procedure in the EU also follows a risk-based approach. However, there are differences in the classification of medical devices (and biomaterials) between the US and the EU. In the EU, this approach uses a set of criteria that can be combined in various ways in order to determine classification, such as duration of contact with the body, degree of invasiveness, local vs systemic effect, potential toxicity, the part of the body affected by the use of the device and if the device depends on a source of energy. This results in four risk classes ranging from Class I (low risk) and IIa (low/Moderate risk) and IIb (Moderate/High risk) to Class III (High risk). Additionally, there are subclasses for Class I based on whether they require sterilization (Is) or have a measuring function (Im). Medical devices in Class III are considered high-risk, require a complete and thorough review of their safety and performance during the CE marking process.

This difference in classification is one example of the challenges faced the evaluation of medical devices in general and biomaterials in particular. Organizations such as the Global Harmonization Task Force (GHTF), now replaced by the International Medical Device Regulatory Forum (IMDRF), have proposed global classification systems that aim to align the risk categorization of medical devices across different regulatory bodies (Schuh and Funk, 2019).

Another challenge is the inconsistency in regulations. The International Organization for Standardization (ISO) authors the major regulatory standards, particularly including the ISO-10993 series, intended for non-clinical biocompatibility and medical device testing. However, national and regional standards supplement these standards, along with independent international and national testing documents. This means that the approval requirements for a specific medical device may vary depending on the regulatory system. For example, an article published in 2018 by Masaeli et al. described that while the FDA may require a multicenter randomized controlled trial with a large patient population for the clearance of a coronary guidewire, the European Regulator may only request a small study without a control group for the same product. These regulatory dissimilarities make the development of medical device in general and biomaterials in particular costly and time-consuming (Masaeli et al., 2019).

In this context, greater harmonization of regulatory standards and processes across different regions and jurisdictions would be highly beneficial. By establishing a more unified and standardized approach to evaluating medical devices and biomaterials, we can reduce regulatory disparities and streamline the approval process. Although international efforts have been initiated to establish common guidelines and frameworks, further progress is required to fully address the need for harmonization.

Furthermore, initiatives like the Medical Device Single Audit Program (MDSAP) promote cooperation among regulatory authorities from different countries (US, Canada, Japan, Brazil, Australia). MDSAP allows for a single audit to be conducted by an authorized auditing organization, which is then accepted by multiple regulatory agencies. This streamlines the auditing process and reduces the burden on manufacturers, enabling them to navigate multiple regulatory systems more efficiently.

In parallel, innovative approaches are being developed to standardize the evaluation of biomaterials. For example, The ClicKit-Well (Fraunhofer IKTS, DE 10 2018 221 415) is an *in vitro* test system that is suitable for direct cell contact studies by allowing size-standardized surface analysis of different materials for quantitative comparison. Complex systems for standardized biomaterials evaluation are being developed too. For instance, PANBioRA biomaterials risk assessment system has been developing a modular apparatus for preclinical material’s evaluation using cutting edge technologies such as cytotoxicity monitoring using sensors (Chmayssem et al., 2021; Chmayssem et al., 2022), organ-on-a-chip models (Huang et al., 2021), and mathematical modelling (Uka et al., 2021; Šušteršič et al., 2021).

## 5 Perspectives

As time goes by, new tools are emerging in the field of biomaterials. New methods allow faster and cheaper development of new biomaterials, but also their better safety evaluation. Among them, machine learning is an approach which uses algorithms that improve upon training on large datasets and is able to find complex patterns, make predictions and decisions. These methodologies have many applications, including biomedical, mostly in the field of genomics (Goecks et al., 2020). Now, it is emerging as a revolutionary tool for faster biomaterials development (Kerner et al., 2021; Suwardi et al., 2022). Recently, we demonstrated, for the first time, utilization of machine learning for prediction of polymer-based coating properties (Gribova et al., 2021; Šušteršič et al., 2023). Other materials properties prediction tools, such as anti-inflammatory, are being developed, and we believe that this trend of *in silico* materials evaluation will continue.

Other innovative systems that are being developed include multicellular and multiple organ systems that will serve to assess new materials safety and functions in a more physiological environment (Picollet-D’hahan et al., 2021; Ahn and Kim, 2021). Modern approaches such as 3D printing and development of microfluidics allow to setup such complex and/or interconnected systems (Carvalho et al., 2018; Goldstein et al., 2021; Jalili-Firoozinezhad et al., 2021). Organ models with incorporated immune, circulatory and nervous system will be developed.

As personalized medicine is being slowly implemented (Mallick, 2021), approaches for personalized testing of new biomaterials will develop too. They can include microbiota-specific infection models, organ-specific inflammation models, as well as models with patient disease status (diabetic, high blood pressure, other metabolic or autoimmune diseases) (Kozjak-Pavlovic et al., 2022; Surolia et al., 2022).

Although many innovative approaches are being and will be developed, their use will require validation and standardization, which will take a significant amount of time until these approaches appear in official regulations. Meanwhile, it remains a responsibility of the researchers to perform a maximum of available preclinical tests to extensively evaluate the new implantable materials, in order to avoid potential complications for the future patients.
